# Weight gain in childhood and blood lipids in adolescence

**DOI:** 10.1111/j.1651-2227.2009.01247.x

**Published:** 2009-06

**Authors:** Bernardo L Horta, Cesar G Victora, Rosângela C Lima, Paulo Post

**Affiliations:** 1 Post-Graduate Programme in Epidemiology, Universidade Federal de PelotasPelotas, RS, Brazil; 2 Universidade Católica de PelotasPelotas, RS, Brazil

**Keywords:** Blood lipids, Catch-up growth, Cholesterol, Cohort

## Abstract

**Aim:**

To assess the effect of weight gain in childhood on blood lipid levels in adolescence.

**Methods:**

A population-based birth cohort carried out in Pelotas, Southern Brazil. All newborns in the city's hospitals were enrolled in 1982. The subjects have been followed up for several times in childhood. At age 18, 79% of all males were followed, and 2083 blood samples were available. Adjusted analyses controlled for household assets index, family income, parental schooling at birth, maternal smoking during pregnancy and breastfeeding duration.

**Results:**

Birth weight for gestational age and weight gain in the first 20 months was not associated with blood lipid levels in adolescence. On the other hand, those subjects whose weight gain from 20 to 42 months of age was faster than that predicted from birth weight and weight-for-age z-score at the mean age of 20 months had lower high-density lipoprotein cholesterol (HDL) cholesterol [−0.78 (95% confidence interval: −1.28; −0.29)] and higher very low-density lipoprotein cholesterol (VLDL) and low-density lipoprotein cholesterol (LDL)/HDL ratio in adolescence. After controlling for current body mass index (BMI), the regression coefficient for HDL cholesterol decreased from −0.78 mg/dL to −0.29 mg/dL (95% confidence interval: −1.00 to 0.05).

**Conclusion:**

Weight gain from 2 to 4 years is related to an atherogenic lipid profile in adolescence and this association is mediated by current BMI.

## INTRODUCTION

Intrauterine malnutrition and early life growth patterns may result in metabolic and physiological programming with lifelong effects on the risk of selected diseases, including several cardiovascular conditions (1). Raised blood concentration of cholesterol would be a potential mechanism underlying these associations.

Huxley et al. (2) reviewed the evidence linking birth weight and blood lipid levels, concluding that impaired foetal growth had little effect. In another meta-analysis, Lauren et al. (3) reached similar conclusions, but noted that after adjustment for current size, low birth weight became strongly related to total cholesterol, apparently supporting a programming effect of birth weight. However, as pointed by Lucas et al. (4) birth size adjusted for current size is a measure of change in size (centile crossing) between these two ages. Therefore, the observation by Lauren et al. (3) is compatible with a more marked effect of postnatal than of intrauterine growth.

Weight gain up to adulthood has been positively associated with total cholesterol or triglycerides or negatively with high-density lipoprotein cholesterol (HDL) cholesterol (5,6,7). On the other hand, Kajantie et al. (8) reported that slow weight gain during infancy was associated with lower HDL cholesterol and higher non-HDL cholesterol at age 55–70 years. With respect to height gain, Eriksson et al. (9) observed that rapid height gain from 7 to 15 years was related to reduced HDL cholesterol concentration in elderly individuals; whereas Miura et al. (10) observed that total cholesterol at 20 years was inversely correlated with height gain from age 3 to 20 years. In the same way, Skidmore et al. (11) reported that total and low-density lipoprotein cholesterol (LDL) cholesterol at 53 years were inversely related with height at 2 years and height velocity between 15 years and adulthood.

Studies in upper-income countries have observed that small for gestational age (SGA) infants often show rapid growth in the first two years (12). In low and middle-income countries, rapid growth in early childhood may have clear short-term benefits. Victora et al. (13) reported that infants who were born SGA and presented accelerated growth in the first 20 months of life had lower mortality and fewer hospital admissions subsequently. On the other hand, several studies suggest that rapid growth later in childhood might increase the risk of coronary heart disease, hypertension (14) and insulin resistance (15). In view of the conflicting evidence, more studies are needed to assess the pros and cons of rapid growth in infancy, to help quantify the so-called catch-up dilemma (16). In particular, studies should investigate whether there are critical periods in infancy and childhood in which rapid growth is more strongly associated with negative outcomes in adulthood (17). This study was aimed at assessing the effect of rapid growth in different age ranges on blood lipid levels of men belonging to a birth cohort.

## METHODS

The study was carried out in Pelotas (current urban population 320 000) in Southern Brazil. The population is mostly white, of Southern European descent. Like Brazil as a whole, there are wide social inequalities in health in this population. The infant mortality rate in the birth cohort was 38 per thousand live births and at the age of 18 years, 17.4% of all male subjects were overweight (body mass index [BMI] above 25 kg/m^2^).

All 5914 infants born alive in three maternity hospitals in 1982 (over 99% of all births in the city) were recruited and have been followed up on several occasions (18). There were 3037 males and 2877 females. Their mothers were interviewed on socio-economic, demographic and health-related variables. Birth weight was obtained by the hospital staff using paediatric scales that were calibrated weekly by the research team and low birth weight was defined as less than 2500 g. Gestational age was estimated from the mothers' recall of their date of last menstrual period, and those subjects whose gestational age was <37 weeks were considered as preterm. SGA was defined as a birth weight below the 10th percentile for gestational age and sex, according to the reference developed by Williams et al. (19).

In 1984 and 1986, city censuses of over 70 000 households were carried out in search of children born in 1982; 87 and 84% of the original cohort were located, respectively, at the average ages of 20 and 42 months (18). Mothers were interviewed and children were weighed using a portable spring scale (CMS, London, UK) with an accuracy of ±100 g, and their length was measured with portable stadiometers. In 1997, all homes in a 27% sample of the city's census tracts were visited, and 72% of cohort members expected to be living in these tracts were interviewed at the mean age of 14.7 years. Again, subjects were weighed with a portable calibrated scale.

In 2000, all males in the birth cohort were legally required to enlist in the Army. A research assistant was deployed at the recruitment office to interview all draftees and to verify if they belonged to the cohort. The cohort members were then invited to answer a questionnaire and to donate a blood sample. Typically, conscripts had continental-style breakfast at home at around 5:30 am, because they had to arrive at 6:00 am at the Army Base where the exams were carried out. Blood samples were collected by venepuncture between 10:30 am and 12:00 noon, after the whole round of medical, physical and intellectual exams had been completed. Male adolescents who did not attend the Army examination were sought at their last known address and invited to attend an examination at a clinic. Those who still failed to attend were visited at home. Overall, 79% of the cohort subjects were traced at this age. The study was approved by the Ethical Review Board of the Faculty of Medicine of the Federal University of Pelotas, and written informed consent was obtained from participating subjects.

The following outcome variables were studied: total serum cholesterol; very low-density lipoprotein cholesterol (VLDL); LDL; HDL and LDL/HDL ratio. Total and HDL cholesterol, and triglycerides were measured using enzyme methods (Dimension® clinical chemistry system; Dade Behring). LDL cholesterol was estimated using the Friedewald's formula (20) VLDL cholesterol was estimated from triglyceride levels. All levels are expressed in mg/dL, except for the LDL/HDL ratio.

Because the ages of children seen at a given follow-up visit varied slightly, we used standard deviation scores (SDS). The SDS indicates the number of standard deviation of a measurement from the mean for that age and sex. Birth weight for gestational age z-scores was also computed. As Williams et al. (19) did not provide the mean birth weight and standard deviation for each gestational age and sex group, the 50th percentile was used as the mean birth weight, and the standard deviation was estimated by subtracting the 10th from the 50th percentile and dividing by 1.28. Using the 2006 World Health Organization growth standards, z-scores were also calculated for weight or length adjusted for age in the follow-up visits.

The following variables collected in the early phases of the study were considered as potential confounders:

- family income at delivery: total income earned by family members during the month before the interview;
- household assets index (obtained through factor analysis and based on the ownership of household goods) (21);
- parental schooling at delivery: years of schooling completed with success;
- breastfeeding duration: This variable was collected in 1984 and 1986. The earliest available information on breastfeeding cessation was used in order to minimize recall bias.
- maternal smoking during pregnancy (non-smokers, 0–14 or 15 or more cigarettes per day).

Blood lipids were analysed as a continuous outcome. The analyses took into account the correlation between weight gain in subsequent age ranges, as well as regression to the mean, by using conditional growth modelling (22). First, birth weight for gestational z-score was used to predict weight-for-age z-score at 20 months; the residual or difference between actual and predicted weight z-score, for each child, was calculated. The regression equation that assessed the effect of weight gain in the first 20 months on cholesterol included birth weight and this residual. Next, weight-for-age z-score at 42 months was predicted from both birth weight and weight-for-age z-score at 20 months; the equation for cholesterol included birth weight, the weight residual at 20 months and the weight residual at 42 months.

## RESULTS

A total of 2250 subjects were interviewed at the age of 18 years, and 2083 blood samples were available. Added to the 143 cohort males known to have died, they represented 78.9% of all live born boys. Table 1 shows that losses to follow-up were more frequent among boys born to the poorest and the wealthiest families, compared to the middle income group. There were no clear trends in follow-up rates relative to birth weight or weight gain from birth to 20 months.

**Table 1:** Follow-up rates at 18 years of age according to baseline characteristics of the cohort

	Percent located at 18 years of age
Monthly family income (US$)	
≤50	72.7
51—100	80.1
101—300	84.0
301—500	79.3
>500	76.6
Maternal schooling at delivery (years)	
≤4	76.2
5—8	81.7
9—11	75.8
≥12	78.9
Birth weight in grams	
<2500	77.5
2500—2999	78.1
3000—3499	78.7
3500—3999	80.1
≥4000	79.9
Change in weight-for-age z-score from birth to mean age 20 months*	
<−0.67	81.7
−0.66 to 0.66	84.6
>0.67	84.5

* Those infants who were not followed up in the 1984 follow-up visit were excluded.

Boys who were followed up in the army study had prevalence of low birth weight, preterm birth and being SGA age of 5.9%, 5.2% and 15.0%, respectively. Information on gestational age was not available for 429 (19.1%) of the subjects. Table 2 also shows the mean and standard deviation for the blood lipids for the 2083 subjects who consented to provide a blood sample; total cholesterol was measured for all of these, but 24 subjects did not provide the amount of blood needed for the remaining tests, which were thus restricted to 2059 individuals.

**Table 2:** Distribution of sample studied at 18 years of age, according to key characteristics

Sample characteristics	N	Mean (SD)	Prevalence
At birth			
Birth weight (g)	2250	3294.0 (521.5)	
Low birth weight	2250		5.9%
Preterm birth	1821		5.2%
Small for gestational age	1822		15.0%
18-year follow-up visit			
Total cholesterol (mg/dL)	2083	143.1 (29.0)	
HDL cholesterol (mg/dL)	2059	40.1 (9.7)	
LDL cholesterol (mg/dL)	2059	88.2 (25.1)	
VLDL cholesterol (mg/dL)	2059	14.8 (9.6)	
LDL/HDL ratio	2059	2.31 (0.81)	

Table S1 shows the adjusted regression coefficients for the blood lipids, according to a difference of one z-score in birth weight for gestational age, or weight-for-age z-score in childhood and adolescence. Birth weight for gestational age and weight-for-age z-score at mean age of 20 months were not significantly associated with blood lipid levels. On the other hand, HDL cholesterol was inversely related to weight-for-age z-score at 42 months and 15 years of age, whereas, VLDL was positively related to weight-for-age z-scores at the same ages. But, given, the small sample size for the 1997 (mean age of 15 years) visit, the VLDL coefficient was not statistically significant.

Table S2 shows the results of conditional growth modelling, adjusted for possible confounding by socio-economic and maternal characteristics. Weight gain is expressed in z-scores of the difference between actual weight and that predicted from earlier weights. Weight gain in the first 20 months of life was not related to blood lipids at 18 years of age. On the other hand, those subjects whose weight gain from 20 to 42 months of age was faster than that predicted from birth weight and weight-for-age z-score at mean age of 20 months had lower HDL cholesterol and higher VLDL, LDL/HDL ratio at 18 years of age.

Table S3 shows that the effect of weight gain from 20 to 42 months on blood lipids levels was not modified by stunting at 20 months.

## DISCUSSION

The prospective nature of the study, its population basis, the use of standardized methods for birth measurements, gestational age assessment and anthropometric evaluation in infancy reduce the likelihood of selection and information bias. Furthermore, the subjects have been followed since birth, conversely to the majority of the studies that have relied on retrospective information on birth data (3), and lacked information on important confounding factors. Some limitations, however, should be considered. Blood lipids were not collected after a 12-h fast. Typically our samples were collected after a 6-h fast following a continental-type breakfast; nevertheless, serum lipid levels were very similar to results from other Latin American settings (23,24). It was not possible to locate 21.0% of the target population. But the proportions of children with fast growth were similar among those who were located and those in the remainder of the original cohort; it is therefore unlikely that the above results have been affected by selection bias.

On the other hand, we were not able to obtain information on gestational age, for 19.1% of the subjects who consented to donate blood, and these subjects were, therefore, excluded from the analyses. Maternal recall of the date of the last menstrual period was lower among low birth weight infants, if maternal recall was also associated with blood lipid levels, the effect of birth weight for gestational age z-score could have been biased. Because blood lipids at 18 years of age were independent of maternal recall of the data of the last menstrual period, we do not believe that the above results have been due to selection bias.

Other studies have assessed the effect of linear growth on blood lipids (9,10,11). Unfortunately, we were not able to assess the effect of linear growth, as information on birth length was not gathered during the perinatal study.

Weight gain from 20 to 42 months was associated with decreased HDL cholesterol, as well as higher VLDL cholesterol. The relationship between rapid weight gain in later childhood and blood lipids was mainly explained by body composition in late adolescence. For example, after controlling for current BMI, the effect of weighing at 42 months one z-score more than that predicted from birth weight and weight at 20 months on HDL cholesterol decreased from −0.78 mg/dL to −0.29 mg/dL (95% confidence interval: −1.00 to 0.05). Since the magnitude of the effect of weight gain in childhood was strongly reduced after controlling to current BMI, our data suggest that the effect of weight gain in later childhood on blood lipids is mediated by BMI in adolescence. Surprisingly, in another analysis, which included those subjects with information on weight at 20, 42 months and 15 years, weight gain from 42 months to 15 years was not related to blood lipids, in spite of its association with increased fatness in late adolescence (25).

Previous analyses of our cohort show that catch-up growth in early infancy has short-term benefits on morbidity and mortality (13), and on the long run on achieved schooling (26). But on the other hand is associated with increased blood pressure (14) and overweight (27) in adolescence, a finding confirmed by other studies (28). In order to help solve ‘the catch-up dilemma’ (16) more research to quantify the advantages and disadvantages of rapid growth in infancy is needed, and analysis should be stratified according to nutritional status in infancy or childhood.

## SUPPORTING INFORMATION

**Table S1** Adjusted regression coefficients of blood lipids according to birth condition and nutritional status in childhood.

**Table S2** Adjusted conditional growth analyses of blood lipids according to predicted weight at the mean ages of 20 and 42 months.

**Table S3** Adjusted regression coefficients of blood lipids by weight gain from 2 to 4 years, according to height-for-age z-score at mean age of 20 months, for all subjects examined at 18 years of age. Each cell represents a regression model.
