# Supplementary material for: Weight gain in childhood and blood lipids in adolescence
Source: Acta Paediatr. 2009 Jun;98(6):1024–8. doi: 10.1111/j.1651-2227.2009.01247.x (PMC2688671; doi:10.1111/j.1651-2227.2009.01247.x)
Supplement: Supplementary file 1 [file apa0098-1024-SD1.doc]

Table S1. Adjusted# regression coefficients of blood lipids according to birth condition and nutritional status in childhood.

|  | | Outcomes | | | | | N |
| --- | --- | --- | --- | --- | --- | --- | --- |
|  |  | HDL | LDL | Total cholesterol | VLDL | LDL/HDL ratio |  |
| Birthweight for gestational age z-score | Coefficient (95% confidence interval) | -0.24  (-0.72; 0.23) | -0.69  (-1.95; 0.58) | -0.85  (-2.29; 0.59) | 0.16  (-0.32; 0.64) | 0.002  (-0.04; 0.04) | 1552 |
|  | P-value | 0.32 | 0.29 | 0.25 | 0.12 | 0.92 |  |
| Weight for age z-score at mean age of 20 months | Coefficient (95% confidence interval) | -0.27  (-0.69; 0.15 ) | -0.56  (-1.66; 0.53) | -0.52  (-1.77; 0.73) | 0.33  (-0.09; 0.74) | 0.014  (-0.02; 0.05) | 1913 |
|  | P-value | 0.20 | 0.31 | 0.42 | 0.12 | 0.45 |  |
| Weight for age z-score at mean age of 42 months | Coefficient (95% confidence interval) | -0.60  (-1.04; -0.16) | -0.57  (-1.72; 0.59) | -0.21  (-1.52; 1.10) | 0.82  (0.38; 1.25) | 0.032  (-0.006; 0.07) | 1788 |
|  | P-value | 0.008 | 0.34 | 0.75 | < 0.001 | 0.10 |  |
| Weight for age z-score at mean age of 15 years | Coefficient (95% confidence interval) | -0,98  (-1,78; -0.19) | 0.63  (-1.35; 2.61) | 0.79  (-1.47; 3.06) | 0.77  (-0.03; 1.57) | 0.071  (0.01 0.13) | 468 |
|  | P-value | 0.015 | 0.53 | 0.49 | 0.06 | 0.03 |  |

# adjusted for household assets, family income, parental schooling at birth and maternal smoking during pregnancy.

Table S2. Adjusted* conditional growth analyses of blood lipids according to predicted weight at the mean ages of 20 and 42 months.

|  | | Outcomes | | | | | N |
| --- | --- | --- | --- | --- | --- | --- | --- |
|  |  | HDL | LDL | Total cholesterol | VLDL | LDL/HDL ratio |  |
| Weight at 20 months minus predicted weight (Z-scores) ^&^ | Coefficient (95% confidence interval) | -0.34  (-0.83; 0.16) | -0.77  (-2.14; 0.45) | -0.84  (-2.38; 0.70) | 0.33  (-0.20; 0.85) | 0.011  (-0.03; 0.06) | 1449 |
|  | P-value | 0.18 | 0.20 | 0.28 | 0.22 | 0.62 |  |
| Weight at 42 months minus predicted weight (Z-scores) ^$^ | Coefficient (95% confidence interval) | -0.78  (-1.28; -0.29) | 0.07  (-1.22; 1.36) | 0.49  (-0.99; 1.97) | 1.18  (0.68; 1.68) | 0.05  (0.01; 0.09) | 1449 |
|  | P-value | 0.002 | 0.92 | 0.52 | < 0.001 | 0.03 |  |

* Adjusted for: household assets, family income, maternal schooling, maternal smoking during pregnancy and breastfeeding duration.

& Also adjusted for birthweight

$ Also adjusted for birthweight and weight residual at 20 months.

Table S3. Adjusted* regression coefficients of blood lipids by weight gain from 2 to 4 years, according to height for age z-score at mean age of 20 months, for all subjects examined at 18 years of age. Each cell represents a regression model.

|  | | Outcomes | | | | | N |
| --- | --- | --- | --- | --- | --- | --- | --- |
|  |  | HDL | LDL | Total cholesterol | VLDL | LDL/HDL ratio |  |
| Height for age z-score < - 2 SDS | |  | | | | | |
| Weight at 42 months minus predicted weight (Z-scores) ^$^ | Coefficient (95% confidence interval) | -0.38  (-1.75; 1.00) | -1.90  (-5.26; 1.46) | -1.60  (-5.45; 2.26) | 0.60  (-0.50; 1.69) | -0.02  (-0.12; 0.08) | 192 |
|  | P-value | 0.59 | 0.27 | 0.41 | 0.29 | 0.71 |  |
| Height for age z-score ≥ - 2 SDS | |  | | | | | |
| Weight at 42 months minus predicted weight (Z-scores) ^$^ | Coefficient (95% confidence interval) | -0.85  (-1.37; -0.32) | 0.38  (-1.02; 1.79) | 0.84  (-0.76; 2.45) | 1.29  (0.74; 1.84) | 0.06  (0.01; 0.11) | 1257 |
|  | P-value | 0.002 | 0.59 | 0.30 | < 0.001 | 0.02 |  |
| P-value interaction | | 0.53 | 0.27 | 0.31 | 0.40 | 0.23 |  |

*adjusted for household assets, family income, parental schooling at birth, maternal smoking during pregnancy, and breastfeeding duration.

$ Also adjusted for birthweight and weight residual at 20 months.
